# The Second International African-Caribbean Cancer Consortium for the study of viral, genetic and environmental cancer risk factors

**DOI:** 10.1186/1750-9378-4-S1-S1

**Published:** 2009-02-10

**Authors:** Camille Ragin, Emanuela Taioli

**Affiliations:** 1 Department of Epidemiology, University of Pittsburgh Graduate School of Public Health, PA, USA; 2 The University of Pittsburgh Cancer Institute, Pittsburgh, PA, USA; 3 Department of Epidemiology and Biostatistics, Downstate School of Public Health, State University of New York, USA

## Introduction

The Second International Conference of the African-Caribbean Cancer Consortium (AC3) was held on May 12–13, 2008 at the Wyndham Miami Airport Hotel & Executive Meeting Center in Miami, FL, USA. The conference was organized by the University of Pittsburgh, Graduate School of Public Health and the University of Pittsburgh Cancer Institute.

The AC3 () was established in 2006 for the study of viral, genetic, environmental, and lifestyle risk factors for cancer in populations of African descent [1]. The goals of this year's conference were to provide a forum for the Consortium members to (1) share their research accomplishments (2) Identify areas for further study and (3) build new collaborations. There were 38 attendees that represented institutions from various countries: The Bahamas; Barbados; Guyana; Jamaica; Nigeria, Africa; Trinidad and Tobago; and the United States (US). The attendees had an opportunity to present their research either through oral or poster presentations. Networking and further discussions were facilitated by question and answer periods after each presentation as well as during the session breaks and dinner reception.

## May 12, 2008

The conference began with the welcome and opening presentation by Dr. Camille Ragin, the principal investigator of the AC3, who defined the AC3's purpose, goals, and provided a brief overview of the membership status as well as a summary of the ongoing AC3 research studies. Currently there are 53 AC3 members many of whom are conducting collaborative studies of cancer risk among individuals of African descent. The AC3 is conducting collaborative studies on the etiology of cervical cancer in the Bahamas, Tobago, Jamaica and US while studies in Guyana and Nigeria are planned. Prostate cancer collaborations have been established in the Bahamas, Jamaica, Nigeria and US. Other collaborative studies that assess cancer prevalence, screening, knowledge and perception have also been established or are planned in Guyana, St. Kitts/Nevis, St. Vincent, US Virgin Islands and the US.

### Session I: Prostate Cancer and HHV8 Infections in Populations of African Descent

This first general session was chaired by Dr. Clareann Bunker from the University of Pittsburgh, US and Dr. Robin Roberts from the University of the West Indies, the Bahamas. The session was lead by a keynote presentation by Dr. Folakemi Odedina of Florida A&M University, US, entitled "Tracing the Root of Prostate Cancer Disparities in Black Men: From West Africa to Caribbean Islands to United Kingdom". Dr. Odedina provided a brief history of the Transatlantic Slave Trade, described how various populations of African descent were connected worldwide. She described the current status of prostate cancer research in these populations, highlighting the limited knowledge of prostate cancer burden in West African populations as well as few research studies in African-Caribbean populations. The presentation ended with a charge that corresponds with the primary goal of the AC3 project. In order to address the prostate cancer disparity in Black men, collaborative studies with standardized methods and protocols are needed in order to explore prostate cancer burden and its risk factors among men of African ancestral origin (African-Caribbean, African-American and African). Session I continued with five additional short presentations of research findings by AC3 members [2-6].

### Session II: Cervical and Other HPV-Associated Cancers in Populations of African Descent

The second general session was chaired by Dr. Camille Ragin from the University of Pittsburgh, US and Dr. Norma McFarlane-Anderson from the University of the West Indies, Jamaica. The keynote address was given by Dr. Raleigh Butler from the University of the West Indies, The Bahamas and was entitled: "Fact, Frictions and Fractions, HPV the Bahamian Experience". Dr. Butler presented an overview of the Epidemiology of HPV and related cancers and described the current status of this disease among Caribbean populations. Cervical cancer is the second cause of cancer deaths in the Bahamas, and annual cervical screening is performed in less than 10% of this at risk population. Session II continued with four additional presentations of human papillomavirus (HPV) related studies conducted in Caribbean populations [7-10].

Day one of this conference concluded with two Special Topics sessions. The first, "Sample Collection, Data Sharing and Confidentiality" was facilitated by Dr. Emanuela Taioli from the University of Pittsburgh. The participants defined appropriate protocols for collection and storage of data and samples as well discussed the importance of standardization in multi-centered collaborative studies. The second Special Topic, "Building Infrastructure for Multi-Center Studies" was facilitated by Dr. Judith Jacobson from Columbia University. During this session the participants discussed decisions and steps necessary for obtaining funding and implementing a collaborative study.

The day ended with a dynamic poster session and networking dinner reception, where the participants continued their discussions about their research projects and reconnected with their colleagues and met new ones.

## May 13, 2008

### Session I: Breast Cancer in Populations of African Descent

This session was chaired by Dr. Michael Okobia, from the University of Benin, Nigeria and Dr. Emanuela Taioli from the University of Pittsburgh, US. Dr. Taioli launched the session as the keynote speaker where she presented the descriptive Epidemiology of Breast cancer among individuals of African ancestry; discussed risk factors for this disease and identified the gaps in research for minority populations. Dr. Taioli also presented some of her current research findings from her studies of African American populations. The session ended with three additional research presentations by AC3 investigators [11-13].

The breast cancer session was followed by a Special Topic discussion on Cancer Registration. There were presentations of cancer statistics by Ms. Veronica Roach from *Dr. Elizabeth Quamina Cancer Registry *in Trinidad and Tobago and Dr. Wallis Best-Plummer representing the Guyana Cancer Registry in Guyana. The presenters provided data from their corresponding cancer registry and were later joined by Dr. Michael Okobia, from the University of Benin, Nigeria for a panel discussion about the establishment process of cancer registries and the challenges surrounding cancer registration in developing countries.

The conference concluded with concurrent breakout sessions where AC3 investigators divided into cancer-specific working groups (cervical, breast and prostate). During these sessions, AC3 investigators discussed the status of the ongoing collaborative studies and defined future studies.

## Conference evaluations

All participants were asked to complete evaluation forms for both meeting dates and 95% of the forms were returned. The majority of the participants (65%) reported that, based on their prior knowledge, they learned more after attending the cancer registration session. In general, participants reported that the conference fulfilled their reason for attending (73%). Eight-five percent were satisfied with the conference content, organization (81%), and overall conference experience (85%).

## Competing interests

The authors declare that they have no competing interests.
